# Ocular Toxicity and Mechanistic Investigation for Berberine and Its Metabolite Berberrubine on Zebrafish

**DOI:** 10.3390/molecules30234602

**Published:** 2025-11-30

**Authors:** Ting Liu, Jia Tang, Xinyi Lu, Lu Jiang, Rui Zhang, Miaoqing Zhang, Jingpu Zhang, Danqing Song, Dousheng Zhang, Mingzhe Xu

**Affiliations:** 1 Institute for Food Control, National Institutes for Food and Drug Control, Beijing 102629, China; lutyliu@126.com; 2 Institute of Medicinal Biotechnology, Chinese Academy of Medical Sciences and Peking Union Medical College, Beijing 100050, China; tangjiaworks@163.com (J.T.);; 3 Institute for Drug Control, National Institutes for Food and Drug Control, Beijing 102629, China; 4 School of Pharmaceutical Engineering, Shenyang Pharmaceutical University, Shenyang 110016, China; 5 Division of Science and Technology, Department of Science, Technology and International Cooperation, National Medical Products Administration, Beijing 100037, China

**Keywords:** BBR, BBR metabolites, ocular toxicity, mitochondrial dysfunction, Sirtuin 3

## Abstract

Berberine (BBR) has seen growing application in ophthalmology, yet the ocular toxicity of BBR and its metabolites remains poorly understood. This study aimed to evaluate the ocular toxicity of BBR and its major metabolite M1 and unravel their underlying mechanisms. Ocular toxicity was evaluated in human corneal epithelial cells and wild-type AB zebrafish. Mechanistic studies utilized fluorescence imaging, biochemical quantitative assays, and qPCR analyses in AB zebrafish and transgenic mitochondrial fluorescent zebrafish (strain *Tg(Xla.Eef1a1:mlsEGFP)*). Both BBR and M1 induced significant ocular toxicity across models, with BBR showing higher toxicity than M1. Mechanistic analyses revealed their toxicity stemmed from photoreceptor cell damage and Sirtuin 3 (SIRT3) inhibition, triggering a cascade of pathological events: mitochondrial dysfunction, oxidative stress, autophagic dysfunction, apoptosis, and inflammation. This study provides a reference for individualized risk assessment and clinical management of BBR-based therapies and paves the way for developing BBR derivatives with reduced ocular toxicity.

## 1. Introduction

For decades, conventional synthetic drugs have formed the cornerstone of pharmacological management in ophthalmology, with a well-documented history in the treatment of prevalent ocular conditions such as glaucoma, bacterial conjunctivitis, and age-related macular degeneration [1,2,3]. Representative agents include β-adrenergic antagonists (e.g., timolol) for intraocular pressure reduction, fluoroquinolone antibiotics for bacterial keratitis, and anti-VEGF biologics for neovascular disorders [1,2,3]. Despite their established efficacy, the long-term and broad application of these drugs is frequently hampered by a spectrum of local complications, such as ocular surface irritation and photophobia [4,5]. Moreover, systemic implications, including bronchospasm and bradycardia associated with topical β-blockers, along with the emerging challenge of antimicrobial resistance, further limit their clinical utility and patient adherence [4,5].

In response, natural products have garnered increasing attention for their inherent biocompatibility and multi-target regulatory potential in treating eye diseases. Among these, berberine (BBR), an isoquinoline quaternary ammonium alkaloid extracted from plants of the *Berberidaceae*, *Ranunculaceae*, and other families, has emerged as a promising candidate. Initially used to treat intestinal infections such as bacillary dysentery and gastroenteritis [6], BBR’s diverse pharmacological effects have been gradually uncovered, leading to its current recommendation for the treatment of diabetes, cardiovascular diseases, and various inflammatory conditions [7,8,9]. Notably, BBR has also been shown to ameliorate diabetic retinopathy (DR) [10,11], marking its entry into ophthalmological research. Since then, its application in the ophthalmological field has achieved remarkable progress, with particular potential demonstrated in the treatment of uveitis, retinal diseases, trachoma, and other ocular conditions [12]. In animal models, BBR intervention increases the survival of injured retinal ganglion cells (RGCs), reduces RGC apoptosis, and significantly improves the visual function [13]. Moreover, it could treat thyroid-associated ophthalmopathy (TAO) by regulating the adipogenesis and fibrosis of orbital fibroblasts (OFs) [14]. Mechanistically, studies have revealed that BBR modulates multiple signaling pathways, such as the AMPK/mTOR, PI3K/AKT/NFκB, and MAPK, thereby reducing inflammation and cell apoptosis in eye disease [10,11,13,15].

**Figure 1:**
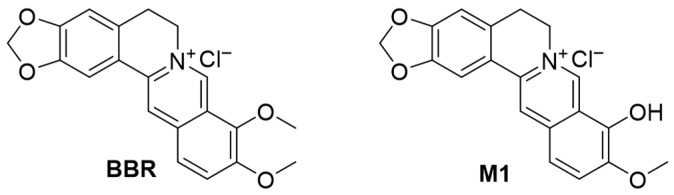
Chemical structures of BBR and M1.

Despite the well-established safety profile of berberine (BBR, Figure 1) when used at normal clinical doses, it may induce specific toxic effects at high doses or with long-term exposure [16,17]. Ocular tissues have a delicate structure and complex functions, rendering them particularly sensitive to drug toxicity; once damaged, the damage is often irreversible. Therefore, the potential ocular toxicity of BBR merits attention. Moreover, orally administered BBR undergoes biotransformation to generate a variety of bioactive metabolites, including berberrubine (M1, Figure 1) with antioxidant, anti-inflammatory, and antitumor effects, demethyleneberberine (M3, Supplementary Figure S1A) with anti-inflammatory potential, and jatrorrhizine (M4, Supplementary Figure S1B) with diverse pharmacological activities [18,19,20]. Although several studies have indicated the potential ocular toxicity of BBR [21,22], there are no systematic studies on that of its metabolites. Additionally, exploring the underlying mechanism of ocular toxicity is also of great importance.

Therefore, this study aims to evaluate the ocular cytotoxicity of four protoberberine compounds with diverse structures, including berberine (BBR) and its three metabolites—M1, M3, and M4. First, BBR and M1 with the most significant cytotoxicity on human corneal epithelial (HCE-T) cell lines were selected to explore their ocular toxicity in zebrafish embryos. Subsequently, the underlying mechanism of their ocular toxicity was explored.

## 2. Results

### 2.1. Cytotoxicity of BBR and Its Analogs on HCE-T Cell Line

**Figure 2:**
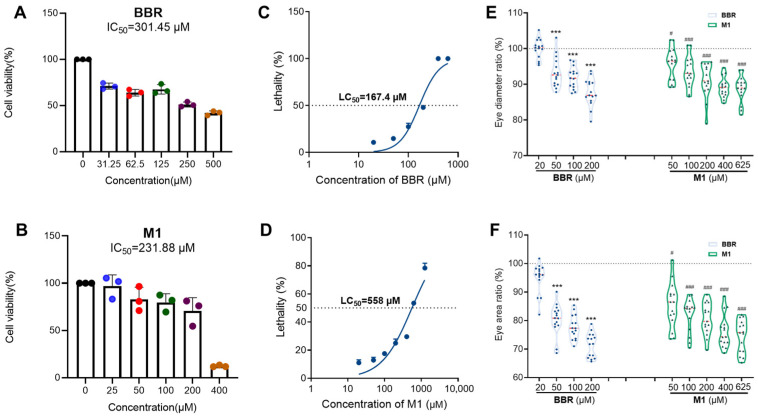
Toxicity evaluation of BBR and M1. (**A**) General toxicity of BBR at concentrations of 31.25, 62.5, 125, 250, and 500 µM on human corneal epithelial cell lines HCE-T; (**B**) general toxicity of BBR at concentrations of 20, 50, 100, 200, and 400 µM on Zebrafish larvae; (**C**) general toxicity of M1 at concentrations of 25, 50, 100, 200, and 400 µM on HCE-T; (**D**) general toxicity of M1 at concentrations of 20, 50, 100, 200, 400, and 625 µM on Zebrafish larvae; (**E**,**F**) ocular toxicity of BBR (**E**) and M1 (**F**) on zebrafish larvae. In vivo observation of ocular phenotypes in zebrafish larvae treated with BBR at concentrations of 20, 50, 100, and 200 µM, or M1 at concentrations of 20, 50, 100, 200, 400, and 625 µM, taking 0.05%DMSO as the control (*n* = 30 per treatment, three independent experiments). The ratio is calculated by dividing the eye diameter or area in BBR or M1 treatment by those in control group. Compared with the control group, the BBR-treated group showed a significant difference, *** *p* < 0.001; and the M1-treated group also showed a significant difference, # *p* < 0.05,### *p* < 0.001.

As the outermost layer of the eye, the corneal epithelium is the first barrier against exogenous drugs and the most direct target of ocular toxicity [23]. Therefore, we initiated the cytotoxicity assay of BBR and its metabolites on HCE-T cells by MTT assay. As shown in Figure 1, BBR and M1, exhibited concentration-dependent cytotoxicity, with IC_50_ values of 301.45 and 231.88 µM, respectively (Figure 2A,B). In contrast, M3 and M4, gave the IC_50_ values of over 400 µM (Supplementary Figure S1C,D). Therefore, both BBR and M1 warranted further investigation.

### 2.2. Ocular Toxicity Evaluations of BBR and M1 in Zebrafish

#### 2.2.1. General Toxicity of BBR and M1 in Zebrafish

The toxicity evaluation of BBR and M1 was subsequently conducted on a zebrafish model featuring mammal-like retinas and transparent embryos [24]. First, the half-lethal concentration (LC_50_) values of berberine (BBR) and berberrubine (M1) in zebrafish were calculated. Wild-type AB strain zebrafish embryos staged at 6 h post-fertilization (hpf), corresponding to the 50% epiboly stage, were exposed to BBR or M1 at different concentrations. Mortality was recorded via stereomicroscopy at 5 days post-fertilization (5 dpf). BBR exhibited significantly higher toxicity, with an LC_50_ value of 164.7 µM, whereas M1 had an LC_50_ value of 558 µM (Figure 2C,D).

#### 2.2.2. Ocular Toxicity of BBR and M1 in Zebrafish

Simultaneously, changes in ocular phenotypes, including eye diameter and eye area were observed using a stereomicroscope, taking 0.05% DMSO as the control. As shown in Figure 2E,F and Supplementary Figure S2, compared with the control group, both BBR and M1 significantly and dose-dependently reduced the eye length and eye area of zebrafish, and BBR exhibiting higher toxicity than M1. These results indicate that both BBR and M1 induce ocular damage in zebrafish larva.

#### 2.2.3. Effects of BBR and M1 on the Locomotor Behavior of Zebrafish Larvae

**Figure 3:**
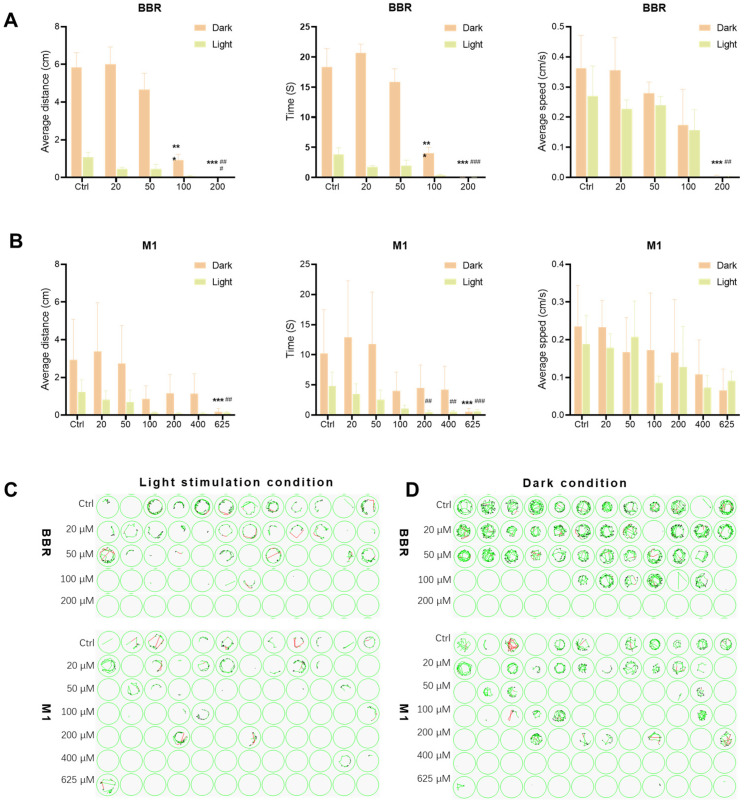
Effects of BBR and M1 on zebrafish behavior. (**A**,**B**) Statistical results of swimming distance, swimming duration, and average swimming speed of larvae under dark stimulation and light stimulation in the BBR (**A**) or M1 (**B**) group (*n* = 12 per treatment, three independent experiments) compared with the control group (0.05% DMSO) under dark stimulation, * *p* < 0.05,** *p* < 0.01, *** *p* < 0.001; compared with the control group under light stimulation, # *p* < 0.05,## *p* < 0.01, ### *p* < 0.001. (**C**,**D**) Effects of BBR and M1 on the swimming trajectories of larvae in the first 30 s of exposure to light stimulation (**C**) and at the last 30 s of dark stimulation (**D**).

We then assessed the locomotor behaviors, including distance, time, and speed of the wild-type AB strain zebrafish larvae under dark and light conditions using an automated tracking system. As shown in Figure 3A, under dark conditions, BBR significantly reduced swimming distance and duration at concentrations above 100 μM and decreased average speed at 200 μM. Upon light stimulation, larvae treated with 200 μM BBR showed significant reductions in all three locomotor metrics. These results indicate that BBR markedly inhibits larval locomotion at high concentrations. In contrast, M1 did not affect locomotion in the dark, but reduced average swimming distance at 625 μM and decreased swimming duration at concentrations above 200 μM (Figure 3B).

We also analyzed larval trajectories under dark conditions and in response to sudden light exposure (Figure 3C,D). In the dark, only minor trajectory deviations were observed in treated larvae, suggesting no major impairment of motor function. However, upon light stimulation, both BBR- and M1-treated larvae exhibited irregular and disorganized swimming patterns compared to controls. Although no statistically significant differences were detected in the quantitative metrics, the altered swimming pattern indicated a reduced ability to regulate movement in response to light. Based on these observations, we hypothesize that these behavioral abnormalities might result from drug-induced ocular damage. This damage may induce photophobia, thereby impairing the larvae’s ability to regulate their responses to light stimuli, which deserves further investigation.

### 2.3. BBR and M1 Induce Apoptosis in Ocular Cells

**Figure 4:**
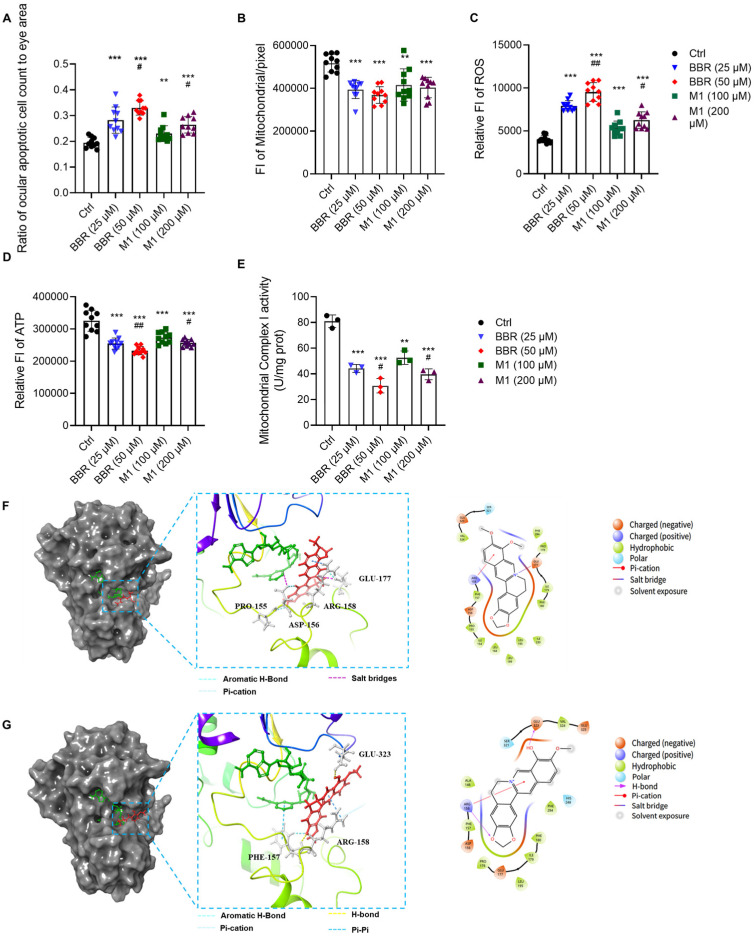
Underlying mechanism of BBR and M1’s ocular toxicity in zebrafish model. (**A**) Proapoptotic effect of BBR and M1 on the eyes of wild-type AB zebrafish (*n* = 30 per treatment, 3 independent experiments). Values are mean ± SD of 10 repeated experiments, randomly selected from the 30 larvae. The ratio is calculated by dividing the fluorescence value of apoptotic intensity in zebrafish ocular cells by the eye area. Given that zebrafish eye size exerts a substantial influence on the data, this method enables us to exclude this confounding factor. (**B**) BBR and M1 induce mitochondrial dysfunction on the transgenic mitochondrial fluorescent zebrafish (strain *Tg(XIEeF1a1:mlsEGFP)*) embryos. Values are mean ± SD of 10 repeated experiments. (**C**,**D**) Effects of BBR or M1 on ROS level and ATP level on the wild-type AB zebrafish (*n* = 30 per treatment, three independent experiments). Values are mean ± SD of 10 repeated samples; each sample contains 3 larvae. (**E**) Effects of BBR or M1 on mitochondria complex I activity on the wild-type AB zebrafish (*n* = 3 per treatment, three independent experiments). Values are mean ± SD of 3 repeated experiments; each sample contains 3 larvae. In the above experiments, 0.05% DMSO was used for the control. Compared with the control group, ** *p* < 0.01, *** *p* < 0.001; compared with the lower dose group of BBR or M1, # *p* < 0.05, ## *p* < 0.01; (**F**) binding pockets of BBR and SIRT3 (PDB code: 5H4D), predicted by Schrödinger Maestro 13.8; (**G**) binding pockets of M1 and SIRT3.

To investigate the underlying mechanism of ocular toxicity, we evaluated the proapoptotic effects of BBR and M1 on wild-type AB strain zebrafish ocular cells. Zebrafish embryos at 6 hpf were water-soluble administration with BBR (25 or 50 μM) or M1 (100 or 200 μM), and the fluorescence intensity of apoptotic ocular cells was measured at 5 dpf using confocal fluorescence microscopy (Figure 4A). Apoptotic cell counts were significantly higher in embryos treated with 50 μM BBR than in those treated with 25 μM BBR. Specifically, the fluorescence intensity (a marker of apoptotic cell abundance) was increased from 0.19 to 0.28 and 0.32 upon the treatments of 25 μM BBR and 50 μM BBR, respectively. Similarly, embryos exposed to 200 μM M1 showed a significant increase in apoptosis compared with those treated with 100 μM M1. Of note, the extent of apoptosis induced by 25 μM BBR was comparable to that caused by 100 μM M1.

Given the central role of mitochondria in energy metabolism and their involvement in apoptosis, we further assessed mitochondrial dynamics change in the transgenic mitochondrial fluorescent zebrafish (strain *Tg(XIEeF1a1:mlsEGFP)*) embryos [25] (Figure 4B). Confocal imaging revealed a decrease in the mitochondrial density of the 5-dpf larvae treated with both BBR and M1. Consistent with the apoptosis results, embryos exposed to higher concentrations of BBR or M1 exhibited significantly more severe damage on the mitochondrial function.

Mitochondrial damage often leads to electron transport chain (ETC) dysfunction, which is characterized by elevated reactive oxygen species (ROS) and reduced ATP production [26]. We therefore examined intracellular ROS and ATP levels in the treated embryos. Intracellular ROS was quantified using the DCFH-DA oxidation assay, a widely used method for detecting ROS in live cells [27]. As shown in Figure 4C, both BBR and M1 treatment resulted in a marked and dose-dependent increase in ROS levels relative to the control. Embryos treated with higher concentrations of either compound showed significantly greater ROS accumulation. In line with these findings, intracellular ATP levels were significantly reduced following BBR or M1 exposure (Figure 4D). Moreover, the higher concentrations of BBR or M1 led to a more pronounced decline in ATP.

### 2.4. BBR and M1 Inhibit the Activity of Mitochondrial Complex I

Inhibition of mitochondrial complex I (NADH dehydrogenase) enzymatic activity is a key upstream cause of mitochondrial dysfunction [28]. Consistent with the previous finding [29], BBR dose-dependently suppressed complex I activity (Figure 4E). Similarly, M1 treatment also induced a marked, dose-dependent decrease in its activity, with higher concentrations of either compound leading to a more significant reductio.

Then, we sought to investigate potential direct interactions between the test compounds and complex I. LibDock analysis (Discovery Studio 4.5) using the complex I structure (PDB code: 6ZKC) revealed no strong binding for either BBR or M1 (Supplementary Figure S3A,B), supporting an indirect mechanism, consistent with the previous finding that BBR inhibits complex I function without directly suppressing its enzymatic activity [29]. Instead, BBR has been found to target Sirtuin 3 (SIRT3), triggering complex I dissociation and thereby impairing mitochondrial activity [29]. We therefore turned to exploring their interaction with SIRT3 (PDB code: 5H4D). Since NADPH acts as a coenzyme to facilitate the function of SIRT3 [29], we applied to cocrystal NADPH-SIRT3 as the protein model to perform the docking analysis via Schrödinger Maestro 13.8. The results (Figure 4F,G) showed that both BBR and M1 integrated well into the same hydrophobic cavity of SIRT3, with the binding scores of −7.810 and −6.879 kcal/mol respectively, indicating BBR exerted a stronger interaction with SIRT3 than M1 did. The planar structure of BBR enabled it to stabilize its binding conformation by forming pi-cation interactions with GLU177 and aromatic hydrogen bonds with ARG158 via hydrophobic pockets. In contrast, M1 adopted a more distorted conformation and primarily formed hydrogen bonds with PHE157 and GLU323. Notably, the advantage of BBR stems from its more favorable hydrophobic interactions, coupled with lower steric hindrance, enabling it to more readily enter the binding pocket. Furthermore, RMSD calculations confirmed that both complexes maintained good stability, with values not exceeding 0.4 nm (Supplementary Figure S4). These results indicate that BBR might exhibit a higher affinity for SIRT3 than M1, consistent with the higher ocular toxicity of BBR.

### 2.5. BBR and M1 Disrupt Zebrafish Genome Maintenance and Functions

To investigate the mechanisms underlying BBR- and M1-induced ocular damage, photophobia, and mitochondrial dysfunction, we employed quantitative PCR (qPCR) to analyze the expression of genes related to photoreceptor cells, oxidative stress, mitochondrial function, and the resultant apoptosis.

**Figure 5:**
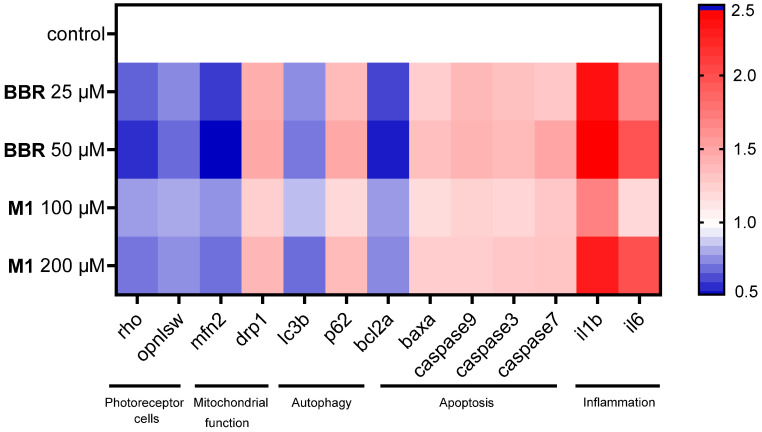
Each value in the heat map represents the ratio of the mean gene expression level of each sample to the mean of the control group. Red series (value > 1) represent upregulated gene expression, white series represent no significant change in gene expression, and blue series (value < 1) represent downregulated gene expression; the depth of red or blue corresponds to the extent of upregulation or downregulation of gene expression.

The *rho* (encoding Rhodopsin) and *opn1sw* (encoding Short-wavelength-sensitive opsin) genes play crucial roles in the zebrafish visual system, with abnormalities in their function closely linked to eye damage [30]. qPCR analysis revealed that both BBR and M1 reduced the expression of these two genes in a dose-dependent manner (Figure 5 and Supplementary Figure S5A). Furthermore, the inhibitory effect of 50 μM BBR was even stronger than that of 200 μM M1, consistent with the more severe ocular damage induced by BBR, as observed above. Notably, their alterations in these two genes indicated that they directly impair ocular function, which may contribute to their impact on the locomotor behavior of zebrafish larvae.

Next, we investigated BBR and M1’s influence on the expressions of two core mitochondrial dynamics genes *mfn2* and *drp1*, which maintain mitochondrial functional homeostasis by mediating mitochondrial fusion and fission, respectively [31]. As disclosed in Figure 5 and Supplementary Figure S5B, BBR and M1 decreased the expression of *mfn2* dose-dependently, while increasing that of *drp1* in a concentration-dependent manner, indicating a disruption on the mitochondiral fusion-fission balance, leading to mitochondrial fragmentation.

Next, we analyzed the changes in the expression of autophagy markers *lc3b* and *p62*. As shown in Figure 5 and Supplementary Figure S5C, BBR and M1 triggered an elevation in *p62* levels while decreasing the expression of *lc3b*. This result suggests the inhibition of damaged mitochondrial clearance by autophagy disorder. Subsequently, we quantified the expression changes of apoptosis-related genes, including *baxa*, *bcl2a*, *caspase9*, *caspase3*, and *caspase7*. BBR and M1 dose-dependently reduced *bcl2a* expression while increasing the expression of *baxa*, *caspase9*, *caspase3* and *caspase7* (Figure 5 and Supplementary Figure S5D), indicating the induction of cell apoptosis. Furthermore, BBR and M1 promoted the transcription of proinflammatory cytokine genes *il1β* and *il6* (Figure 5 and Supplementary Figure S5E), suggesting the induction and exacerbation of inflammatory responses.

## 3. Discussion

Ocular toxicity represents a critical aspect of drug safety evaluation, directly impacting patients’ visual health and quality of life. BBR has gained widespread attention in ophthalmic therapy, with accumulating evidence supporting its efficacy in treating trachoma, diabetic retinopathy, age-related macular degeneration (AMD), glaucoma, and uveitis [10,11,12]. In contrast, the ocular toxicity profile of berberine (BBR) remains incompletely characterized despite its promising therapeutic potential, highlighting the need for a systematic safety evaluation. Existing studies have reported fragmented results. In rabbit models, topical application of 1.5 mg/mL BBR delayed corneal epithelial repair [32]. Beyond the cornea, BBR also exhibits phototoxic effects on other ocular cell types: 25 μM BBR under UVA exposure caused severe damage to human lens epithelial cells (HLE-B3) [33].

Notably, the clinically used concentrations of BBR in ophthalmic formulations vary widely. Anti-infective BBR eye drops typically contain 0.1–1.0% (*w*/*v*), equivalent to 300–3000 μM [34], while ocular protective formulations use considerably lower levels, around 0.01–0.5% (*w*/*v*) or 30–1500 μM [35]. Crucially, no previous study has systematically assessed BBR toxicity across a broad concentration range in corneal epithelial cells—the primary barrier for topically applied drugs. To address this gap, we evaluated BBR at 0–500 μM for in vitro cytotoxicity, 0–200 μM in zebrafish mortality assays, and 50 μM and 100 μM for mechanistic investigations. Our results identify BBR-induced toxicity at 50 μM, offering critical insights for defining safety thresholds in human ophthalmic formulations.

Moreover, the ocular toxicity of BBR metabolites and derivatives remains unreported, representing a major gap in the overall safety assessment. This study systematically evaluates BBR and its major metabolite M1 across multiple experimental models, thereby complementing existing data and establishing a solid foundation for the safe clinical development of BBR-based ocular therapies.

Our study demonstrates that both BBR and its metabolite M1 induce significant ocular developmental toxicity in zebrafish embryos. Both compounds produce consistent phenotypic alterations, characterized by dose-dependent reductions in eye size parameters, establishing a clear dose–effect relationship. Furthermore, exposed larvae exhibited distinct behavioral deficits, including reduced locomotor activity, disordered swimming trajectories, and diminished responsiveness to light stimulation, alongside alterations in the expression of genes *rho* and *opn1sw*, collectively suggesting functional impairment secondary to structural ocular damage.

The eye is particularly vulnerable to disruptions in energy and oxidative homeostasis. This study provides the first evidence in zebrafish that berberine (BBR) inhibits mitochondrial complex I activity in ocular tissues. As a pivotal enzyme in the respiratory chain, the inhibition of complex I disrupts electron transport, leading to reduced ATP production and increased electron leakage, which in turn generates excessive reactive oxygen species (ROS). This energy disruption effect of BBR is closely associated with the suppression of SIRT3 [29]. Our qPCR results demonstrated that BBR and its metabolite M1 induced a mitochondrial dynamics imbalance, characterized by the downregulation of *mfn2* and upregulation of *drp1*. This was accompanied by impaired autophagy, as evidenced by decreased *lc3b* and increased *p62* expression. Furthermore, these changes activated the apoptotic pathway, indicated by a decreased *baxa/bcl2a* ratio and elevated levels of *caspase9*, *caspase3*, and *caspase7*. Concurrently, an inflammatory response was triggered, marked by upregulation of *il1β* and *il6*.

Extensive published evidence confirms that inhibition of mitochondrial complex I is largely attributable to SIRT3 suppression, and aggravated apoptosis and mitochondrial dysfunction are specifically driven by SIRT3 inhibition. These studies have utilized multiple approaches, including SIRT3 knockout/knockdown models [36], SIRT3-specific activators/inhibitors [37,38], and direct complex I activity measurements [29], to demonstrate that SIRT3 suppression directly impairs complex I function by regulating the acetylation status of its subunits. Mechanistically, SIRT3 inhibition or deficiency directly downregulates mitofusin 2 (MFN2) and upregulates dynamin-related protein 1 (DRP1), resulting in mitochondrial fragmentation and amplified apoptotic signaling. This specificity is corroborated by rescue experiments, where restoration of SIRT3 reverses the MFN2/DRP1 imbalance and abrogates apoptosis [39,40]. In contrast, targeting other Sirtuin family members or oxidative stress pathways (e.g., the Nrf2/HO-1 axis) does not recapitulate this regulatory cascade [41,42].

**Figure 6:**
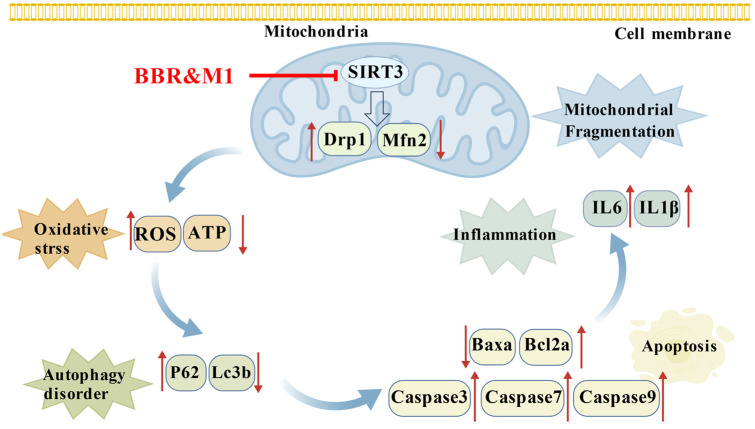
Potential mechanism of ocular toxicity of BBR and M1 in zebrafish [44].

Therefore, the cascading events collectively form an interconnected pathological network (Figure 6): Inhibition of SIRT3 by BBR and M1 induces mitochondrial dynamics imbalance, which directly triggers oxidative stress and ATP depletion, key mediators linking dynamics imbalance to subsequent damage. This damage is then amplified by autophagic impairment, followed by apoptosis activation that executes cell death, and inflammatory responses that further exacerbate tissue injury. Within this network, SIRT3 suppression acts as the molecular trigger, driving mitochondrial fragmentation and excessive ROS production. These outcomes not only reflect upstream signaling defects but also directly contribute to downstream autophagic dysfunction, which in turn serves as a critical hub aggravating the pathological process. Owing to the failure in clearing damaged organelles, apoptosis and inflammation are simultaneously activated, ultimately culminating in an irreversible cell fate decision [43].

Moreover, a key comparative finding reveals BBR’s superior ocular toxicity over M1. While sharing structural similarity, their differential C9 substituents (-OCH_3_ in BBR vs. -OH in M1) significantly influence physicochemical properties. BBR, with a higher affinity to SIRT3, raised higher ocular toxicity than M1 This mechanistic understanding positions M1 formation as a detoxification pathway, providing scientific rationale for establishing appropriate impurity thresholds in BBR-based pharmaceuticals and highlighting the necessity of metabolite evaluation in comprehensive safety assessments.

Our study has several limitations that should be acknowledged. The behavioral abnormalities (e.g., reduced locomotor activity, impaired light response) induced by BBR or M1 are attributed to structural ocular damage, supported by evidence of photoreceptor cell impairment. However, we have not ruled out non-ocular contributions, such as systemic mitochondrial dysfunction affecting neural function, that may also drive these behavioral deficits.

## 4. Materials and Methods

### 4.1. Chemicals

BBR and M4 were purchased from Shanghai Aladdin Biochemical Technology Co., Ltd. (Shanghai, China), while M1 and M3 were synthesized in-house and stored in our compound library. The purity of all compounds was ≥95%, as determined by HPLC.

### 4.2. Cell and Cell Culture

The human corneal epithelial cell line HCE-T (Cellosaurus accession: CVCL_1272, RRID: CVCL_1272, RCB: RCB2280) was purchased from Procell Life Science and Technology Co., Ltd. (Wuhan, China). DMEM cells were cultured in high-glucose Dulbecco’s Modified Eagle Medium (DMEM), which was purchased from Procell Life Science and Technology Co., Ltd. (Wuhan, China). The high-glucose DMEM was DMEM basic medium supplemented with 15% fetal bovine serum (Procell), 1% penicillin/streptomycin (Procell), 5 μg/mL insulin, and 10 ng/mL human epidermal growth factor (Procell).

### 4.3. MTT Assay

HCE-T cells were seeded in 96-well plates (8 × 10^3^ cells/well, 100 μL medium) and cultured overnight at 37 °C, 5% CO_2_ for adhesion. BBR or its metabolites at various concentrations (BBR and M3 at the concentrations of 31.25, 62.5, 125, 250 and 500 µM, M1 and M4 at the concentrations of 25, 50, 100, 200 and 400 µM) in 0.5% DMSO were added and incubated for 24 h, with 0.5% DMSO in culture medium as the vehicle control. Subsequently, 20 μL MTT (5 mg/mL) was added to each well and the cells were cultured at 37 °C for 4 h. Then, the culture medium was removed, and 150 μL DMSO was added to dissolve formazan crystals and the absorbance at 570 nm was measured on a Synergy H1 multifunctional microplate reader (Biotek, Winooski, VT, USA). The IC_50_ values of test compounds were calculated by Graphpad Prism 8.5 (GraphPad Software Co., San Diego, CA, USA).

### 4.4. Zebrafish Models

The adult zebrafish (Danio rerio) AB strain was obtained from Dr. Anming Meng (Tsinghua University, Beijing, China) and preserved at the Institute of Medicinal Biotechnology, Chinese Academy of Medical Sciences or IACUC Chairman of Hunter Biotechnology, Inc. (Hangzhou, China). The relevant genetic information of this strain is deposited in public databases, with the ZFIN (Zebrafish Information Network) ID: ZDB-GENO-960809-7 and NCBI BioProject accession number: PRJNA482893. Transgenic mitochondrial fluorescent zebrafish (strain *Tg(Xla.Eef1a1:mlsEGFP)*); note: corrected to the standard nomenclature “Xla.Eef1a1”, referring to Xenopus laevis elongation factor 1 alphawere obtained from Zhejiang Ringbio Biotechnology Co., Ltd. (Beijing, China). Its genetic information is available in authoritative databases, including ZFIN ID: ZDB-TGCONSTRCT-090309-1 and CZRC (China Zebrafish Resource Center) ID: CZ222. 

Zebrafish embryos and larvae were maintained in 24-well plates (10 embryos per well) in fish-rearing water at 28 °C with a 14-h light/10-h dark cycle [25]. The water quality parameters were as follows: 200 mg of instant sea salt was added to 1 L of reverse osmosis water, with an electrical conductivity of 450–550 μS/cm, a pH of 6.5–8.5, and a water hardness of 50–100 mg/L CaCO_3_.

In the following experiments, wild-type AB strain zebrafish embryos were used, except for the mitochondrial function assay, where *Tg(Xla.Eef1a1:mlsEGFP)* strain zebrafish were employed instead. Embryos at 6 hpf (50% epiboly stage) were grouped and exposed to incubation solutions of berberine (BBR) at concentrations of 20, 50, 100, 200, and 400 µM, or M1 at concentrations of 20, 50, 100, 200, 400, and 625 µM, until 5 dpf. The control group was treated with 0.05% DMSO in saline. All experiments were performed in three biological replicates; unless otherwise specified, each replicate included 30 larvae per treatment, resulting in a total of 90 larvae per treatment.

### 4.5. Zebrafish Embryonic Developmental Toxicity

Wild-typed AB strain zebrafish embryos at the 6 h post-fertilization stage (6 hpf) were grouped (30 fishes per group), and BBR at the concentrations of 20, 50, 100 and 200 µM or M1 at the concentrations of 20, 50, 100, 200, 400 and 625 µM were administered by microinjection, taking 0.05% DMSO in saline as the control. The mortality was recorded everyday by SZX2 stereomicroscopy (Olympus, Hachioji-shi, Japan) until 5 dpf. Dead embryos were recorded and discarded every 24 h. The experiment was performed three times using 30 embryos per concentration (*n* = 90 per group in total). Using the mortality rates recorded at 5 dpf across all test concentrations, LC_50_ values were determined via Graphpad Prism 8.5 (GraphPad Software Co.; San Diego, CA, USA), which employs a standard method for calculating median lethal concentrations in toxicological assays.

### 4.6. Zebrafish Eye Length and Area Experiment

Half of the epiboly embryos (6 hpf under standard conditions) were exposed to solutions (2 mL) of BBR at the concentrations of 20, 50, 100, and 200 µM or M1 at the concentrations of 20, 50, 100, 200, 400, and 625 µM and cultured under standard conditions, taking 0.05% DMSO in saline as the control. The experiment was performed three times using 30 embryos per concentration (*n* = 90 per group in total). At 5 dpf, embryos were washed 3× with normal medium, transferred to fresh medium, and photographed by E3ISPM20000KPA digital camera (ToupTek, Hangzhou, China), with the magnification of 10–40× and exposure time of 200–500 ms. Then the eye length and area were measured and analyzed using Image J V2.1.X (NIH, Bethesda, MD, USA). Data (eye length/area) were expressed as mean ± SD, compared with the control group, and plotted.

### 4.7. Zebrafish Visual Motor Response Experiment

Wild-type larvae (6 hpf, 30 per group) were incubated with BBR at the concentrations of 20, 50, 100, and 200 µM or M1 at the concentrations of 20, 50, 100, 200, 400, and 625 µM for 5 days. At 120 hpf, larvae were tested in a 96-well plate (1 larva/well, 12 per group). After 10 min acclimation, light-dark transition tests were performed (5 min dark to 5 min light, repeated 3 times), with recordings every 1 min for 40 min. The trajectory and 1 min movement distance were recorded by Behavioral Tracking System: Daniovision, Zebralab 3.3 (ViewPoint, Civrieux, France). Data were processed in Excel (Microsoft, Redmond, WA, USA). Metrics included 1 min total distance, movement time, and average speed. Averages of 12 larvae per group were used for bar graphs; comparisons with the control group were analyzed via *t*-test. The experiment was conducted three times in parallel.

### 4.8. Fluorescence Microscopy for Ocular Cell Apoptosis

A total of 30 wild-type AB strain zebrafish at 6 hpf were treated with BBR at 25 or 50 μM, M1 at 100 or 200 μM, or 0.05% DMSO (control), respectively, until 2 dpf, with 30 larvae per treatment and three biological replicates (total 90 larvae per group). The zebrafish larvae were then stained with acridine orange. After staining, 10 larvae were randomly selected from each experimental group and imaged using a motorized-focus continuous-zoom fluorescence microscope AZ100 (Nikon, Shinagawa-ku, Japan) with the magnification of 10–40× and exposure time of 200–500 ms. The fluorescence intensity of ocular apoptotic cells and mitochondria in zebrafish larvae was measured and analyzed using NIS-Elements 6.10 (Nikon Imaging Software, Nikon, Japan).

### 4.9. Fluorescence Microscopy for Mitochondrial Function

Strain *Tg(Xla.Eef1a1:mlsEGFP)* zebrafish larvae (6 hpf) were treated with BBR (25 or 50 μM), M1 (100 or 200 μM), or 0.05% DMSO (control) until 5 dpf, with 30 larvae per treatment and three biological replicates (total 90 larvae per group). Then, 10 larvae were randomly selected from each experimental group and imaged using a motorized-focus continuous-zoom fluorescence microscope AZ100 (Nikon, Japan) with the magnification of 10–40× and exposure time of 200–500 ms. The fluorescence intensity of ocular apoptotic cells in zebrafish larvae and mitochondria throughout the zebrafish were measured and analyzed using NIS-Elements (Nikon Imaging Software, Nikon, Japan). The experiment was conducted three times in parallel.

### 4.10. Determination of Cellular ATP and ROS Levels

Wild-type AB strain zebrafish (6 hpf) were treated with BBR (25 or 50 μM), M1 (100 or 200 μM), or 0.05% DMSO (control) until 5 dpf, with 30 larvae per treatment and three biological replicates (total 90 larvae per group). Subsequently, 3 larvae were pooled per sample (10 samples per group) and the entire zebrafish was transferred directly into a 96-well plate for fluorescence detection. Cellular ATP content was determined using the CellTiter-Glo^®^ Luminescent Cell Viability Assay Kit (Promega, Madison, WI, USA), which relies on a bioluminescent principle (luciferase-luciferin reaction). Cellular ROS levels were measured using CM-H2DCFDA kits (Invitrogen, Carlsbad, CA, USA), based on the oxidation of non-fluorescent CM-H2DCFDA to fluorescent DCF by ROS. Reagents were prepared and reactions were performed according to the manufacturers’ instructions, with luminescence detected using a Spark multifunctional microplate reader (Tecan, Grödig, Austria).

### 4.11. Mitochondrial Complex I Activity Assay

Wild-type AB strain zebrafish (6 hpf) were treated with BBR (25 or 50 μM), M1 (100 or 200 μM), or 0.05% DMSO (control) until 5 dpf, with 9 larvae per treatment and three biological replicates (total 18 larvae per group). Three zebrafish larvae were harvested per group (3 samples per group) and homogenized on ice following the protocol of the Mitochondrial Respiratory Chain Complex I Activity Assay Kit (Solabor, Beijing, China). Briefly, cell homogenates were centrifuged at 600× *g* for 5 min at 4 °C, and the supernatants were transferred to new microcentrifuge tubes. The samples were then centrifuged again at 11,000× *g* for 10 min at 4 °C; the precipitates (containing mitochondria) were used for the complex I activity assay. These precipitates were subjected to ultrasonication in the appropriate reaction buffer, and reactions were set up according to the protocol. After mixing, the OD_340_ values at time 0 (OD1) and at 2 min (OD2) were recorded using a Synergy H1 multifunctional microplate reader (Biotek, Winooski, VT, USA). The ΔOD value was calculated by subtracting OD2 from OD1. Mitochondrial complex I activity was calculated according to the formula provided in the protocol and expressed as units per mg of sample protein.

### 4.12. RNA Extraction and Reverse Transcription

Wild-type AB strain zebrafish (6 hpf) were treated with BBR (25 or 50 μM), M1 (100 or 200 μM), or 0.05% DMSO (control) until 5 dpf, with 30 larvae per treatment and three biological replicates (total 90 larvae per group). A total of 30 larvae were pooled per sample (3 samples per group), collected, and homogenized. Total RNA was isolated using a commercial RNA extraction kit following the manufacturer’s protocol. The concentration and purity of the extracted RNA were determined by measuring the absorbance at 260 nm and 280 nm using a NanoDrop 2000 ultraviolet-visible spectrophotometer (Thermo, Waltham, MA, USA). A total of 1 µg of RNA from each sample was reverse-transcribed into complementary DNA (cDNA) using a PrimeScript RT reagent kit (Takara Bio Inc., Dalian, China). The reverse transcription reaction was performed in a T100 Thermal Cycler (BIO-RAD, Singapore) under the following conditions: 37 °C for 15 min, followed by 85 °C for 5 s.

### 4.13. Quantitative Real-Time PCR (qPCR)

The qPCR reactions were carried out to quantify the relative expression levels of target genes, including photoreceptor-related genes (*rho* and *opn1sw*), mitochondrial dynamics genes (*mfn2* and *drp1*), autophagy markers (*lc3b* and *p62*), apoptosis-related genes (*baxa*, *bcl2a*, *caspase9*, *caspase3*, and *caspase7*), and inflammation-related genes (*il1b* and *il6*), on a CFX Connect quantitative real-time PCR instrument (BIO-RAD, Singapore). The experiment was performed according to the manufacturer’s instruction. β-actin served as the internal reference, and the experiment was repeated in triplicate.

### 4.14. Molecular Docking

The molecular docking between BBR or M1 with 3D structures of complex I (PDB code: 6ZKC) was carried out using the Libdock module of Discovery Studio 4.5 (BIOVIA, Paris, France). Before docking, protein structure and ligands were minimized.

Docking simulations of BBR or M1 with SIRT3 (PDB code: 5H4D) were performed on Schrödinger 13.8 (Schrödinger, New York, NY, USA). First, BBR and M1 were prepared via the software’s LigPrep module, with pH adjusted to 7.4 ± 0.05 to mimic physiological conditions. Induced Fit Docking was subsequently employed for docking assays, with all other parameters set to default. 

### 4.15. Statistical Analysis

Values are presented as mean ± standard deviation (SD) of 3 or 10 repeated samples. For most zebrafish experiments, 90 larvae per group were employed, based on considerations of statistical power (medium effect size, α = 0.05, power ≥ 80%), field practices, and practical feasibility [45]. The Graphpad Prism software versions 8.5 and 10.8 (GraphPad Software Co.; San Diego, CA, USA) were used for statistical evaluation. Quantitative data are analyzed by one-way analysis of variance (ANOVA) followed by Student–Newman–Keuls test for multiple comparisons, and enumeration data are analyzed by X2 test. *p* < 0.05 is considered to be statistically different.

## 5. Conclusions

To conclude, both BBR and its metabolite M1 induce significant, dose-dependent toxic effects on human corneal epithelial cells, as well as on ocular development in zebrafish embryos. They markedly induce zebrafish eye damage, as evidenced by reduced eye length and eye area, thereby impairing the swimming trajectories of zebrafish larvae and their ability to respond to light stimuli. Mechanistically, we demonstrate that the ocular toxicity stems from photoreceptor cell damage and SIRT3 inhibition, which triggers a cascade of interconnected pathological events involving mitochondrial dysfunction, oxidative stress, autophagic dysfunction, apoptosis, and inflammation. Notably, metabolite M1 causes less severe ocular damage compared to BBR, highlighting a potential direction for developing less toxic analogs. These findings not only clarify the mechanism underlying BBR’s ocular effects but also underscore the importance of assessing metabolite safety. Clinically, caution should be exercised when administering BBR, particularly in patients with retinal or mitochondrial disorders, given its potential ocular toxicity.

## Data Availability

The original contributions presented in this study are included in the article/Supplementary Materials. Further inquiries can be directed to the corresponding authors.

## Supplementary Materials

The following supporting information can be downloaded at: https://www.mdpi.com/article/10.3390/molecules30234602/s1, Figure S1: Chemical Structure and Toxicity Evaluation of M3 and M4. Figure S2: Ocular toxicity of BBR and M1 to zebrafish larvae. Figure S3: Binding mode between BBR, M1 and Complex I structure. Figure S4: Dynamic simulation of the optimal BBR or M1-SIRT3 pose. Figure S5: Gene Expression and Validation.

## Abbreviations

The following abbreviations are used in this manuscript:

BBR	berberine
SIRT3	Sirtuin 3
DR	diabetic retinopathy
RGCs	retinal ganglion cells
TAO	thyroid-associated ophthalmopathy
OFs	orbital fibroblasts
M1	berberrubine
M3	demethyleneberberine
M4	jatrorrhizine
PAL	palmatine
DPAL	dehydroxypalmatine
CBBR	cycloberberine
STE	short-term exposure
LC_50_	half-lethal concentration
ETC	electron transport chain
ROS	reactive oxygen species
qPCR	quantitative real-time PCR
OCTs	organic cation transporters
