# Supplementary material for: Ocular Toxicity and Mechanistic Investigation for Berberine and Its Metabolite Berberrubine on Zebrafish
Source: Molecules. 2025 Nov 30;30(23):4602. doi: 10.3390/molecules30234602 (PMC12693135; doi:10.3390/molecules30234602)

Supporting Information

Ocular Toxicity and Mechanistic Investigation for Berberine and Its Metabolite Berberrubine on Zebrafish

Ting Liu [1,†], Jia Tang [2,†], Xinyi Lu [3,4], Lu Jiang [5], Rui Zhang [2], Miaoqing Zhang [2], Jingpu Zhang [2], Danqing Song [2], Dousheng Zhang [3,*] and Mingzhe Xu [3,*]

1 Institute for Food Control, National Institutes for Food and Drug Control, Beijing 102629, China; lutyliu@126.com
2 Institute of Medicinal Biotechnology, Chinese Academy of Medical Sciences and Peking Union Medical College, Beijing 100050, China; tangjiaworks@163.com (J.T.)
3 Institute for Drug Control, National Institutes for Food and Drug Control, Beijing 102629, China
4 School of Pharmaceutical Engineering, Shenyang Pharmaceutical University, Shenyang 110016, China
5 Division of Science and Technology, Department of Science, Technology and International Cooperation, National Medical Products Administration, Beijing 100037, China
* Correspondence: zhangds@nifdc.org.cn (D.Z.); xumzhe@nifdc.org.cn (M.X.); Tel.: +86-1053851544 (D.Z.); +86-1053851375 (M.X.)
† These authors contributed equally to this work.

Legends

Figure S1 Chemical Structure and Toxicity Evaluation of M3 and M4.

Figure S2 Ocular toxicity of BBR and M1 to zebrafish larvae.

Figure S3 Binding mode between BBR, M1 and Complex I structure.

Figure S4 Dynamic simulation of the optimal BBR or M1-SIRT3 pose.

Figure S5 Gene Expression and Validation.

Figure S1 Chemical Structure and Toxicity Evaluation of M3 and M4. A) Chemical structure of M3; B) chemical structure of M4; C) general toxicity of M3 at concentrations of 31.25, 62.5, 125, 250, and 500 μM on human corneal epithelial cell lines HCE-T; D) general toxicity of M4 at concentrations of 25, 50, 100, 200, and 400 μM on HCE-T.

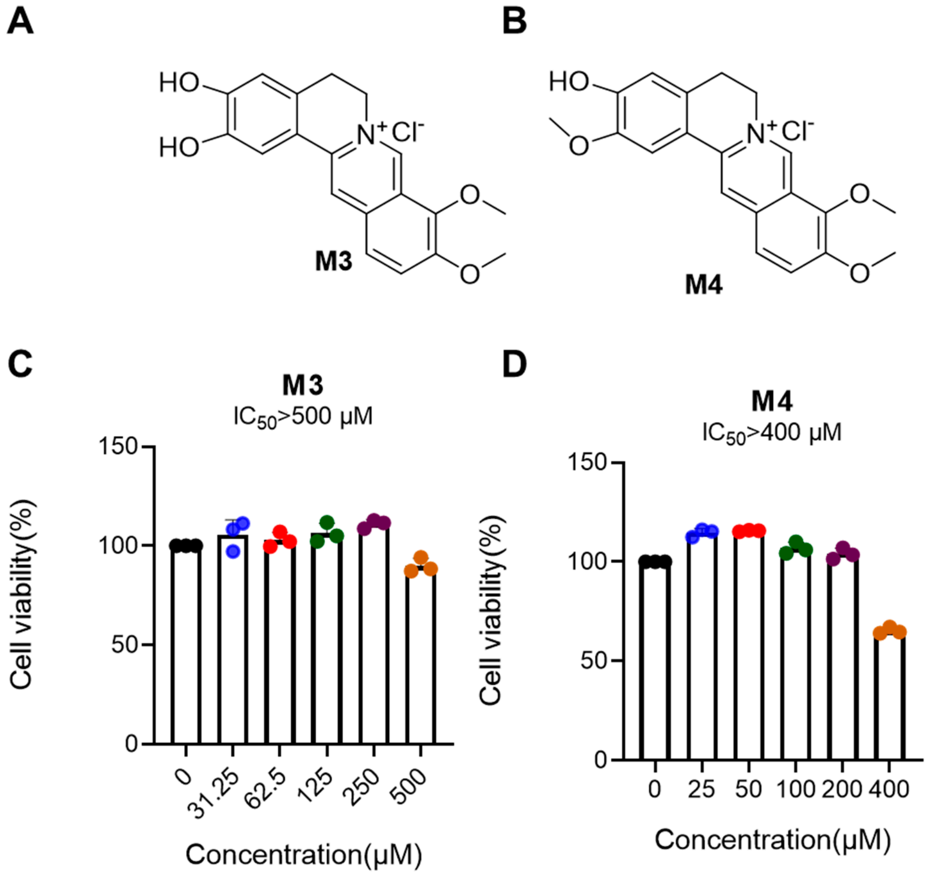

Figure S2 Ocular toxicity of BBR and M1 to zebrafish larvae. In vivo observation of ocular phenotypes in zebrafish larvae treated with different concentrations of BBR and M1 taking 0.05% DMSO as the control (n = 30 per treatment, three independent experiments)

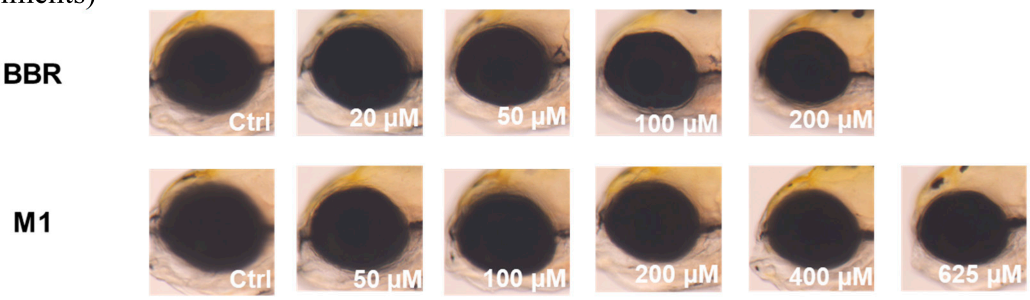

Figure S3 Binding mode between BBR, M1 and Complex I structure. A) LibDock analysis using the complex I structure (PDB code: 6ZKC; predicted by Libdock Discovery Studio 4.5) revealed no strong binding for either BBR or M1. B) An example of no direct binding.

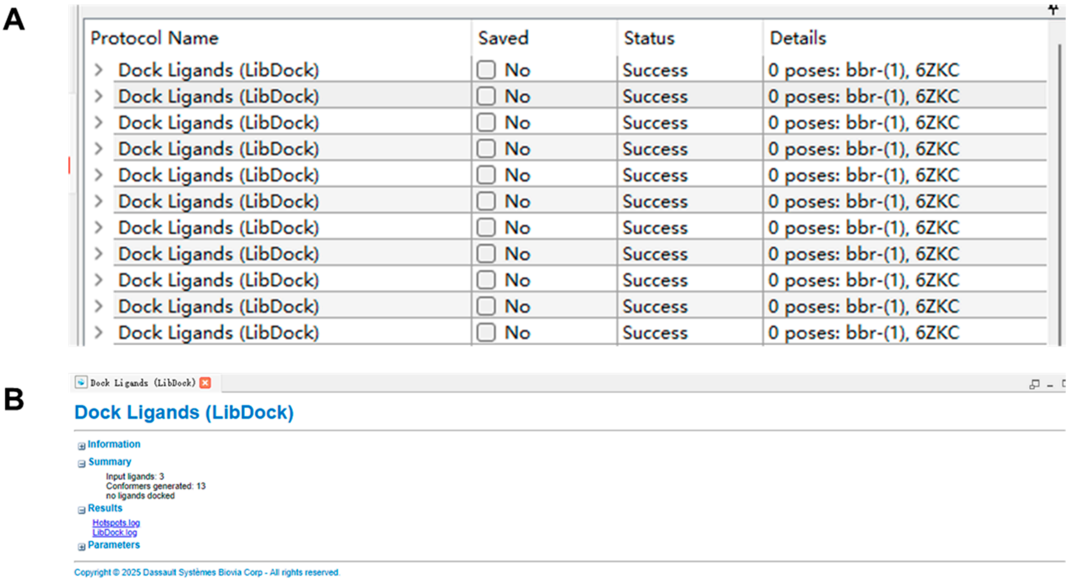

Protocol Name	Saved	Status	Details
Dock Ligands (LibDock)	No	Success	0 poses: bbr-(1), 6ZKC
Dock Ligands (LibDock)	No	Success	0 poses: bbr-(1), 6ZKC
Dock Ligands (LibDock)	No	Success	0 poses: bbr-(1), 6ZKC
Dock Ligands (LibDock)	No	Success	0 poses: bbr-(1), 6ZKC
Dock Ligands (LibDock)	No	Success	0 poses: bbr-(1), 6ZKC
Dock Ligands (LibDock)	No	Success	0 poses: bbr-(1), 6ZKC
Dock Ligands (LibDock)	No	Success	0 poses: bbr-(1), 6ZKC
Dock Ligands (LibDock)	No	Success	0 poses: bbr-(1), 6ZKC
Dock Ligands (LibDock)	No	Success	0 poses: bbr-(1), 6ZKC
Dock Ligands (LibDock)	No	Success	0 poses: bbr-(1), 6ZKC
Dock Ligands (LibDock)	No	Success	0 poses: bbr-(1), 6ZKC

Figure S4 Dynamic simulation of the optimal BBR or M1-SIRT3 poses. A) Dynamic simulation of the optimal BBR-SIRT3 pose; B) dynamic simulation of the optimal M1-SIRT3 pose.

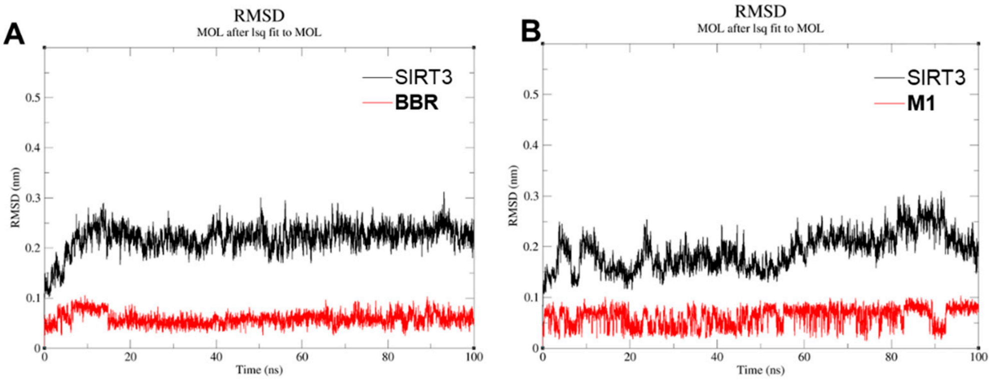

Figure S5 Gene Expression and Validation. BBR and M1 changed the expression levels of A) photoreceptor cell-related genes, including *rho* (encoding rhodopsin) and *opn1sw* (encoding short-wavelength-sensitive opsin); B) mitochondrial fusion/fission genes *mfn2* (encoding mitofusin 2) and *drp1* (encoding dynamin-related protein); C) autophagy-related genes, including *lc3b* (encoding microtubule-associated protein 1 light chain 3β) and *p62* (encoding sequestosome 1, SQSTM1); D) apoptosis-related genes, including *baxa* (encoding BCL2-associated X protein), *bcl2a* (encoding BCL2-associated protein A), *caspase7* (encoding caspase 7), *caspase3* (encoding caspase 3) and *caspase9* (encoding caspase 9); and E) inflammation-related genes, including *il1b* (encoding interleukin-1β) and *il6* (encoding interleukin-6). Compared with the control group, *P < 0.055, **P < 0.01, ***P < 0.001 (n = 3); compared with the lower dose group of BBR or M1, #P < 0.05, ##P < 0.01(n = 3).

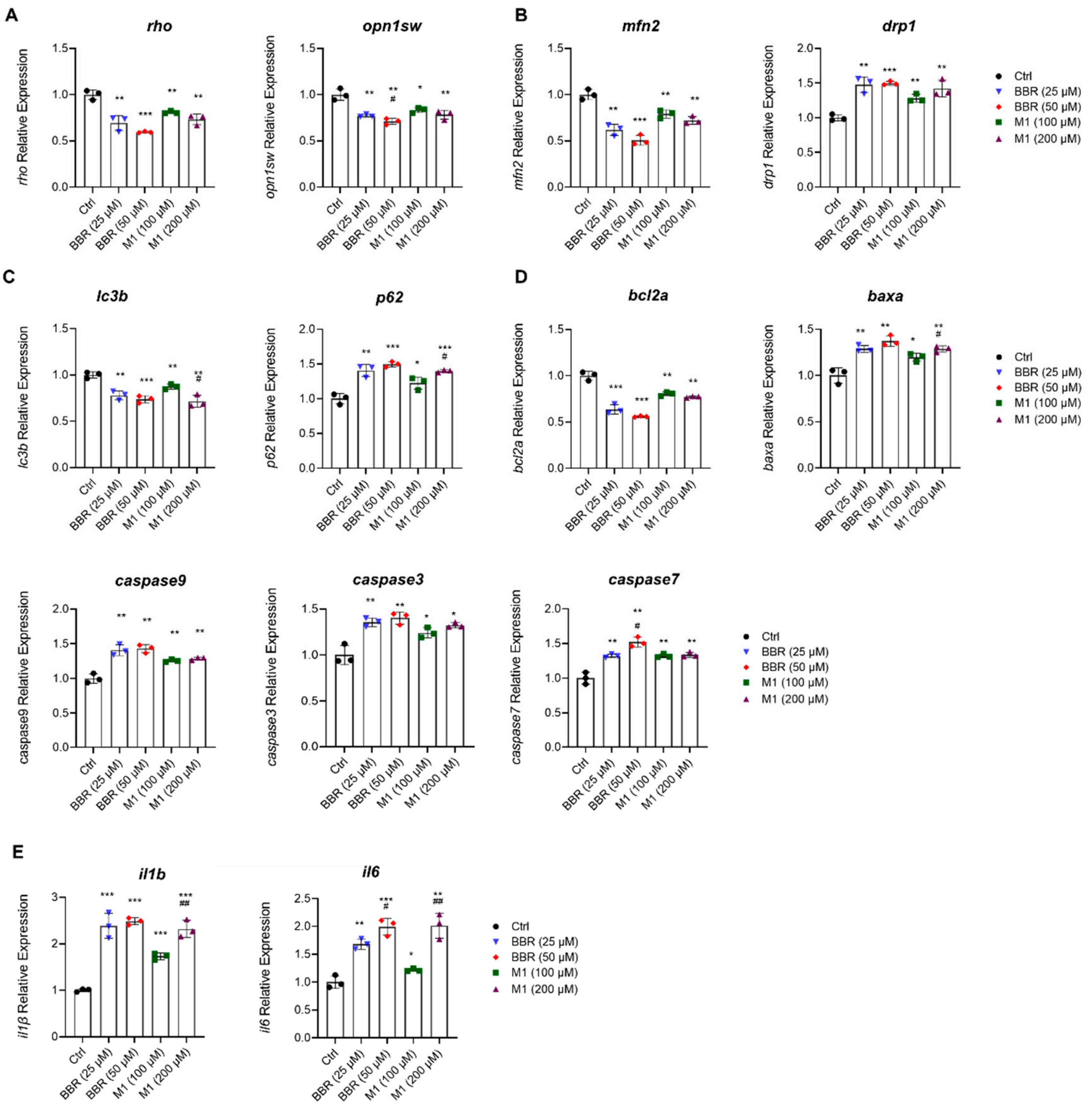

Supplement: Supplementary file 1 [file molecules-30-04602-s001.zip › molecules-3994884-supplementary.pdf]
